# Maternal and foetal medical conditions during pregnancy as determinants of intrapartum stillbirth in public health facilities of Addis Ababa: a case-control study

**DOI:** 10.11604/pamj.2019.33.21.17728

**Published:** 2019-05-14

**Authors:** Alemayehu Gebremariam Agena, Lebitsi Maud Modiba

**Affiliations:** 1 Integrated Nutrition and WASH Program, Kigali, Rwanda; 2 Department of Health Studies, University of South Africa, TvW 7-160 College of Human Sciences, Unisa, South Africa

**Keywords:** Addis Ababa, intrapartum stillbirth, medical condition, maternal, foetal, pregnancy, infection, ANC

## Abstract

**Introduction:**

globally, intrapartum stillbirth accounts for 1 million deaths of babies annually, representing approximately one-third of global stillbirth toll. Intrapartum stillbirth occurs due to causes ranging from maternal medical and obstetric conditions; access to quality obstetric care services during pregnancy; and types, timing and quality of intrapartum care. Different medical conditions including hypertensive & metabolic disorders, infections and nutritional deficiencies during pregnancy are among risk factors of stillbirth. Ethiopia remains one of the 10 high-burden stillbirth countries with estimated rate of more than 25 per 1000 births.

**Methods:**

a case-control study using primary data from chart review of medical records of women who experienced intrapartum stillbirth in 23 public health facilities of Addis Ababa during the period July 1, 2010 - June 30, 2015 was conducted. Data was collected from charts of all cases of intrapartum stillbirth meeting the inclusion criteria and randomly selected charts of controls in two to one (2:1) control to case ratio.

**Results:**

chronic medical conditions including diabetes, cardiac and renal diseases were less prevalent (1%) among the study population whereas only 6% of women experienced hypertensive disorder during the pregnancy in review. Moreover, 6.5% of the study population had HIV infection where being HIV negative was protective against intrapartum stillbirth (aOR 0.37, 95% CI 0.18-0.78). Women with non-cephalic foetal presentation during last ANC visit were three times more at risk of experiencing intrapartum stillbirth whereas singleton pregnancy had strong protective association against intrapartum stillbirth (p<0.05).

**Conclusion:**

untreated chronic medical conditions, infection, poor monitoring of foetal conditions and multiple pregnancy are among important risk factors for intrapartum stillbirth.

## Introduction

Stillbirth is one of the adverse outcomes of pregnancy. The definition of stillbirth can vary from country to country based on clinical and obstetric care protocols. Some literature define stillbirth as a baby born after the 24^th^ week of pregnancy who did not at any time breathe or show any other sign of life after being completely removed from the mother [1]. For international comparisons, WHO recommends reporting of stillbirth with birthweight of 1000 g or more, 28 weeks' gestation or more, or a body length of 35cm or more, commonly reported as third-trimester stillbirth [2]. Furthermore, foetal death occurring during labour is referred as intrapartum stillbirth [3]. Globally, nearly 2.6 million third trimester stillbirth occurred in 2015. Notwithstanding the gestational age cut-off for its definition, stillbirth can occur either during antepartum or intrapartum period. Evidence shows that most of stillbirth can be prevented through the correct application of clinical and obstetric skills hence the current high prevalence is unacceptable. Furthermore, approximately 98% of all stillbirth occur in low and middle-income countries (LMIC), nearly 10-fold higher than those documented in high-resource settings [4]. Stillbirth classification varies along geographic regions, causes and timing of stillbirth in relationship to labour. The older concept of macerated versus fresh stillbirth roughly corresponds to gross categorisation of antepartum and intrapartum categories, but with the advent of ultrasound and foetal heart rate (FHR) monitoring tools, the timing of the stillbirth in developed countries is generally known, at least approximately [5]. Regardless of the classification challenges, intrapartum stillbirth accounts for one million deaths of babies annually, representing approximately one-third of global stillbirth toll. These estimates highlight the magnitude of loss of life just minutes and hours prior to birth with devastating social, emotional and epidemiologic consequences [6].

Moreover, literature shows that stillbirth in general and intrapartum stillbirth particularly occurs due to attributable underlying causes ranging from maternal medical and obstetric conditions; access to quality obstetric care services during pregnancy; and types, timing and quality of intrapartum care. Ethiopia remains one of the 10 high-burden countries with estimated rate of more than 25 per 1000 births [4]. The Ethiopian Demographic and Health Survey (2011) reported 46 perinatal deaths per 1000 total birth annually where Addis Ababa experienced approximately 30 per 1000 births for the same period [7]. Medical conditions of the mother during the time of each pregnancy can determine pregnancy outcomes. Marshall and Raynor (2014:224) describe different medical conditions including hypertensive, metabolic, endocrine, respiratory, haematological disorders, infections and nutritional deficiencies that can emerge or become aggravated during pregnancy as critical factors that could cause adverse pregnancy outcomes including stillbirth [8]. For instance, of the 20,000 pregnancies that resulted in stillbirth (39% intrapartum stillbirth) in South Africa between 2008-2009, 20% were associated with hypertensive disease that could have been managed to avert the adverse outcomes [9]. Similarly, HIV and syphilis infections are widely believed to have statistically significant associations with stillbirth. For instance, a study from Namibia reported that approximately 26% of cases of stillbirth in the study population had history of HIV infection during their index pregnancies [10]. A study from North-Eastern Ethiopia showed that pregnant women with syphilis infection were three times more likely to develop stillbirth [11]. Many of these risk factors could be screened and managed as part of the standard antenatal care services, making the latter an indispensable public health practice. Accordingly, this study collected data on key maternal medical conditions including hypertension, diabetes, infections, Antenatal Care (ANC) attendance, and foetal condition during the pregnancy in review from the public health facility in Addis Ababa to see if any of these had statistically significant associations with intrapartum stillbirth compared to the livebirth outcomes.

## Methods

**Study setting and design:** this was a case-control study using primary data from chart review of medical records of women who experienced intrapartum stillbirth in 20 public health centres and 3 public hospitals of Addis Ababa during the period July 1, 2010 - June 30, 2015. In 2010, 26 public health centres offered Basic Emergency Obstetric and Neonatal Care (BEmONC) in Addis Ababa [12] out of which 20 were selected for this study due to service volume. Similarly, chart reviews were conducted in three out of the five public hospitals under the Addis Ababa City Administration, where Comprehensive Emergency Obstetric and Neonatal Care (CEmONC) had been practiced since 2010. Therefore, this study was conducted in a health-facility setting with intrapartum stillbirth as an outcome of interest.

**Sampling:** all cases of intrapartum stillbirth that occurred in the public health facilities in Addis Ababa were recorded in the maternity registers which is the sampling frame for this study. Given intrapartum stillbirth is a relatively rare phenomenon, this study included all cases of intrapartum stillbirth meeting the inclusion criteria and recorded in the maternity care registers in 20 public health centres and three hospitals between July 1, 2010 - June 30, 2015. Controls were selected from the same maternity registers which helped as sampling frame in each public health facility using a lottery method and in two to one (2:1) control to case ratio. Therefore, in each facility, two medical charts of women with livebirths were selected for each case of intrapartum stillbirth. On every page of the maternity registers where cases of intrapartum stillbirth were taken, record numbers of women with livebirth were listed and rolled on pieces of paper of which an individual other than the data collector randomly selected the required number of controls.

**Sample size:** accordingly, of the documented 112 intrapartum stillbirth cases in the 20 public health centres in Addis Ababa, 91 (81%) met the selection criteria and were included in this study. Similarly, there were a total of 944 cases of intrapartum stillbirth in the three public hospitals of which 637 (67%) qualified the inclusion criteria. A total of 427 charts of controls were reviewed in the 20 public health centres of which only 273 (64%) were included. Moreover, 1738 controls were also randomly identified in the three public hospitals in the city of which 1278 (74%) qualified the inclusion criteria. In general, 728 cases of intrapartum stillbirth and 1551 controls were considered from all the target public health facilities in Addis Ababa. Quantitative data on key variables related to maternal medical conditions that are considered risk factors to intrapartum stillbirth were collected from maternal ANC follow up and obstetric records of women who had given birth in the public health facilities in Addis Ababa from Jul 1, 2010 - June 30, 2015. Data entry and analysis were conducted using SPSS version 24 from August 1 - Sept 30, 2016. Bivariate analysis was conducted for key independent variables followed by multivariate logistic regression model for variables with p-value of 0.2 and less.

**Ethical Considerations:** data was collected from medical records thereby minimising the concerns of confidentiality and requirements for individual consents. The data collector was trained and strictly monitored on the principles of confidentiality of clients' information during the process of data collection. The chart review was conducted within the respective facilities through consented authorisation of relevant facility leadership. Individual data sources remained anonymous during analysis and report presentation. Furthermore, ethical approval was obtained from the Higher Degrees of the University of South Africa (HSHDC/421/2015) and study permit was secured from health ethics committee of Addis Ababa Regional Health Bureau (AARHB) prior to data collection.

## Results

**Socio-demographic characteristics:** data was collected on five key socio-demographic variables including age, marital status, gravida, parity and number of children alive for cases whose charts were reviewed. Accordingly, approximately 57% of women who experienced intrapartum stillbirth and 60% who had livebirths reported to be in the age category 25-34 years. The second highest proportion of women in the study population for both intrapartum stillbirth (35.8%) and livebirth (33.6%) were found in the age group 15-24 years. Results from this study showed that proportionally more women in the intrapartum stillbirth category (49.3%) than in the livebirth (37.1%) conceived for the first time. Consistent with the results on gravida, intrapartum stillbirth was proportionally more common among primigravida (60%) compared to those who given birth to up to three children. This study did not reveal any statistically significant differences between intrapartum stillbirth and livebirth categories for women of three and higher birth orders. However, the descriptive results from this study showed that women with one or more alive children were proportionally less likely to experience intrapartum stillbirth compared to women without any child (Table 1).

**Table 1:** key socio-demographic characteristics against intrapartum stillbirth

Characteristic	Stillbirth N (%)	Live birth N (%)	p-value
**Age (Years)**			
15-24	261(35.8)	522(33.6)	0.333
25-34	416(57.2)	931(60.3)	
35-49	51(7.0)	98(6.1)	
**Marital Status**			
Married	314(42.7)	982(64.4)	0.386
Divorced	3(0.4)	5(0.3)	
Widowed	0(0.0)	3(0.2)	
Separated	0(0.0)	2(0.1)	
Never Married	11(1.5)	43(2.8)	
Missing	400(54.9)	516(33.2)	
**Gravida**			
One	360(49.3)	575(37.1)	0.000
Two	203(28.0)	539(34.8)	
Three	84(11.5)	256(16.5)	
Four	55(7.6)	133(8.6)	
Five and Above	26(3.7)	48(3.0)	
**Parity**			
Zero	442(60.3)	744(48.1)	0.000
One	185(25.4)	542(35.0)	
Two	57(7.9)	177(11.4)	
Three	31(4.3)	61(3.9)	
Four	10(1.5)	19(1.2)	
Five and Above	4(0.5)	8(0.5)	
**Children**			
Zero	451(68.8)	790(55.2)	0.000
One	134(20.4)	435(30.4)	
Two	43(6.6)	139(9.7)	
Three	21(3.2)	49(3.4)	
Four and Above	7(1.1)	17(1.2)	
Missing	72(9.8)	121(7.8)	

**Maternal medical condition:** only 6.3% of women in the intrapartum stillbirth category and 6.1% of women in the livebirth category reportedly had higher blood pressure during the pregnancies in this study. Similarly, prevalence of other common maternal medical conditions including diabetes, cardiac and renal disease were less than 1% for both groups. On the contrary, 90% and 93% cases and controls were HIV negative during the pregnancy in review respectively. Furthermore, approximately 82% of cases against 91% controls tested negative for syphilis among the study population. The prevalence of syphilis was 0.7% and 0.8% among cases and controls respectively. Considerably high amount of data was missing for both cases (17%) and controls (8.4%) which shows poor record keeping practice and limited diagnostic procedures in the public health facilities of Addis Ababa. Results from the current study showed that proportionally more women in the livebirth (91.9%) category than stillbirth (87.7%) were Rh+, which was protective compared to being Rh-ve during pregnancy. Ironically, there were slightly more Rh-ve women in the livebirth category than stillbirth however referring to the larger missing data among stillbirth group (7.7%) than livebirth (2.9%), the protective association of being Rh+ among livebirth category seems justifiable.

**Foetal medical condition during pregnancy:** foetal risk factors including foetal heart rate, foetal presentation and the presence of multiple pregnancy during the last ANC visit in the public health facilities were also analysed to see their relevance to intrapartum stillbirth. Over 97% of women in both stillbirth and livebirth categories had normal FHR during the antenatal visit for the pregnancies in review. Proportionally more women in the intrapartum stillbirth category (10.7%) than in livebirth group (3.7%) had non-cephalic presentation of foetus during the last ANC visit of the pregnancy in reference. Similarly, proportionally more women in the intrapartum stillbirth group (6.5%) than in the livebirth (3.7%) had multiple babies during the pregnancy in review (Table 2).

**Table 2:** distribution of maternal medical history during the pregnancy in review

Characteristics	Stillbirth N (%)	Live birth N (%)	p-value
**Hypertension**			
Yes	46(6.3)	94(6.1)	
No	682(93.7)	1448(93.9)	
Missing	0	9	0.880
**Diabetes**			
Yes	2(0.3)	9(0.6)	
No	726(99.7)	1542(99.4)	0.519
**Cardiac disease**			
Yes	0(0.0)	3(0.2)	
No	728(100.0)	1548(99.8)	0.556
**Renal diseases**			
Yes	3(0.4)	3(0.2)	
No	725(99.6)	1548(99.8)	0.397
**Sero-status for HIV infection**			
HIV Positive	48(6.5)	79(5.1)	0.009
HIV Negative	657(90.1)	1440(93.2)	
Don’t Know	29(3.4)	31(1.6)	
**Sero-status for Syphilis**			0.000
Positive	5(0.7)	12(0.8)	
Negative	600(82.3)	1406(90.9)	
Don’t Know	123(17.0)	133(8.4)	
**Blood Group and Rh**			
Positive	643(87.7)	1415(91.9)	0.000
Negative	32(4.4)	80(5.2)	
Don’t Know	63(7.9)	56(2.9)	
**Foetal Heart Rate (FHR)**			
Normal	721(97.8)	1525(98.8)	0.087
Abnormal	0(0.0)	0	
Don’t Know	25(2.2)	26(1.2)	
**Foetal Presentation**			
Vertex	617(83.7)	1420(92.0)	0.000
Breech	76(10.3)	56(3.6)	
Shoulder	3(0.4)	2(0.1)	
Don't know	69(5.6)	73(4.3)	
**Multiple Pregnancy**			
Yes	47(6.5)	57(3.7)	0.010
No	672(92.7)	1459(95.7)	
Don’t Know	9(0.8)	35(0.5)	
**Number of ANC Visits**			
Once	478(65.3)	490(32.0)	0.000
Twice	60(8.2)	180(11.8)	
Three times	52(7.1)	163(10.7)	
Four times and more	142(19.4)	696(45.5)	
Missing	474(65)	512(33)	

## Discussion

This study assessed various risk factors including socio-demographics, maternal medical conditions during the pregnancy in review to determine associations with intrapartum stillbirth. Intrapartum stillbirth was highly concentrated in 15-34 years without any statistically significant difference between cases and controls. This finding is comparable with a study from Kenya [13]. Evidence shows that maternal medical conditions including hypertensive, metabolic, endocrine, respiratory, haematological disorders are associated with adverse pregnancy outcomes including stillbirth [8]. Up to 10% of pregnancies can be affected by hypertensive disorders commonly diagnosed prior to 20 weeks of gestation and these medical conditions can determine intrapartum stillbirth outcome [14-16]. For instance, studies show chronic kidney diseases and diabetes during pregnancy can be associated with stillbirth [17-19]. Findings from this study revealed that many of the chronic medical conditions including diabetes, cardiac and renal diseases were less prevalent (1%) among the study population whereas only 6% of women experienced hypertensive disorder during the pregnancies in review. These findings were not consistent with similar studies probably owing to poor record keeping and limited diagnostic capabilities in the public health facilities in the study setting. For instance, studies in Ethiopia and Kenya reported higher prevalence of hypertension among women of reproductive age 29% and 9.2% respectively [15, 20].

HIV infection during the pregnancies in review had statistically significant associations between case and control categories. To this effect, the 6.5% HIV prevalence among the study population was comparable with a similar finding from Cameroon [21]. Being HIV negative had statistically significant protective association against intrapartum stillbirth (aOR 0.37, 95% CI 0.18-0.78) among the study population. This finding was consistent with studies from Namibia and Zambia where HIV infection was reportedly significantly associated with stillbirth [10, 22]. This finding resonates with other similar studies that established statistically significant associations between HIV infection and stillbirth [23, 24]. The prevalence of syphilis was relatively low compared to another study in the same context, which reported 2.9% among pregnant women [11]. Not being infected with syphilis during the pregnancy in review had a protective association against intrapartum stillbirth (p<0.001) however this association was not statistically significant after adjusting for confounders. A study from north-eastern Ethiopia showed that pregnant women with syphilis infection were three times more likely to develop stillbirth [11]. It is imperative that pregnant women are screened for infections early in pregnancy and should receive appropriate treatment to prevent intrapartum stillbirth. Furthermore, data on three important foetal risk factors including foetal heart rate, foetal presentation and the presence of multiple babies during the last ANC visit of pregnancies in review were analysed in this study. Evidence shows that FHR and foetal presentation may not be good predicators until third trimester however late ANC visits can identify potential risks associated with these factors [25]. This study revealed that pregnant women with non-cephalic foetal presentations during last ANC visits of the index pregnancy were three times more at risk of experiencing intrapartum stillbirth compared to those with cephalic foetal presentations (OR 3.14, 95% CI 2.21-4.46).

A study from Nepal reported that women with non-cephalic presentation were 12 times more likely to experience intrapartum stillbirth [26]. Equally important observation in this current study was the fact that unestablished diagnosis of foetal presentation during late pregnancy had increased the odds of intrapartum stillbirth albeit without any statistical significance (OR 1.47, 95% CI 0.87-2.51). Missed diagnosis of foetal presentation can happen due to combination of factors including limited competence of service providers, absence of technology like ultrasound equipment, poor recording and follow-up of important pregnancy related tests and interventions. Misdiagnosis of important obstetric parameters during last ANC visit and labour can be seen as an indication of poor quality obstetric care. Empirical evidence shows chance of correctly diagnosing non-cephalic foetal presentation particularly among nulliparous and obese women are lower; hence requiring strong competence coupled with diagnostic technologies [27]. The presence of multiple pregnancy during the pregnancy in review was another important variable observed in this study. Over 92% of women in both intrapartum stillbirth and livebirth categories had singleton pregnancy during the last ANC visit that showed strong protective association against intrapartum stillbirth (p<0.05). This result was comparable with a similar study from Ghana where 8.7% of pregnancy with multiple pregnancy had stillbirth [28]. A study from Taiwan also showed that multiple gestations had markedly increased the risk of adverse fatal outcomes including stillbirth [29]. A systematic review also indicated that twin pregnancies are high risk that can results in thirteen-fold increase in the rates of stillbirth (Table 3) [30].

**Table 3:** distribution of key factors associated with maternal and foetal medical conditions during pregnancy

Independent variable	Birth outcome		Crude OR (95% CI)	Adjusted OR (95% CI)
	Stillbirth N (%)	Live birth N (%)		
Gravida *				
One	363 (49.3)	573 (37.1)	1.1 (0.67-1.8)	
Two	206 (28.0)	537 (34.8)	0.67 (0.41-1.1)	
Three	85 (11.5)	254 (16.5)	0.58 (0.34-0.99)	
Four and above	83 (11.3)	179 (11.6)	1	
**Parity**				
Zero	444 (60.3)	741 (48.1)	1.19 (0.35-4.00)	
One	187 (25.4)	539 (35.0)	0.69 (0.21-2.33)	
Two	58 (7.9)	176 (11.4)	0.66 (0.19-2.27)	
Three	32 (4.3)	60 (3.9)	1.06 (0.29-3.82)	
Four and above	15 (2.0)	26 (1.7)	1	
**Children alive**				
Zero	451 (68.8)	790 (55.2)	1.78 (1.47-2.17)**	1.48 (1.12-1.95)**
One or more	205 (31.2)	640 (44.8)	1	1
Sero-status for HIV infection				
HIV positive	48 (6.5)	79 (5.1)	0.60 (0.31-1.18)	0.05 (0.21-1.21)
HIV negative	662 (90.1)	1431 (93.2)	0.46 (0.26-0.81)*	0.37 (0.18-0.78)*
Don’t know	25 (3.4)	25 (1.6)	1	1
**Blood group and Rh**				
Positive	643 (87.7)	1415 (91.9)	1	1
Negative	32 (4.4)	80 (5.2)	0.88 (0.58-1.34)	1.27 (0.71-2.25)
Don’t know	58 (7.9)	45 (2.9)	2.84 (1.90.-4.23)**	1.62 (0.87-3.02)
**Multiple pregnancy**				
Yes	47 (6.5)	57 (3.7)	1.09 (0.35-3.39)	
No	672 (92.7)	1459 (95.7)	0.61 (0.21-1.77)	
Don’t know	6 (0.8)	8 (0.5)	1	
**Sero-status for Syphilis**				
Positive	5 (0.7)	12 (0.8)	0.43 (0.15-1.26)	1.49 (0.41-5.43)
Negative	604 (82.3)	1401 (90.9)	0.46 (0.34-0.58)**	0.99 (0.65-1.50)
Don’t know	125 (17.0)	129 (8.4)	1	1
Foetal Presentation during ANC***				
Vertex	617 (83.7)	1420 (92.0)	1	1
Non-vertex	79 (10.7)	58 (3.8)	3.14 (2.21-4.46)**	1.01 (0.56-1.82)
Don’t know	41 (5.6)	66 (4.3)	1.43 (0.95-2.14)	1.47 (0.87-2.51)

## Conclusion

Maternal and foetal medical conditions during last ANC visit are among avertable drivers of intrapartum stillbirth. Uncontrolled chronic illnesses, infection, poor monitoring of foetal conditions, and multiple pregnancy are risk factors for intrapartum stillbirth. Although data from this study didn't reveal clear association between chronic medical conditions and intrapartum stillbirth, the observed poor record keeping and limited diagnostic capabilities in the public health facilities in the study setting might have contributed to the inconsistence of these finding with similar studies. Strengthening the skills and motivation of obstetric service providers in the public health facilities in Addis Ababa is imperative to reduce intrapartum stillbirth. Furthermore, HIV and Syphilis infection had significant association with intrapartum stillbirth, a situation that needs to be addressed through correct screening and timely treatment of the conditions prior or during early pregnancy. Appropriate and close monitoring of foetal risk factors including foetal heart rate, foetal presentation, and the presence of multiple pregnancy particularly in the third trimester can prevent intrapartum stillbirth in resource limited settings including the public health facilities of Addis Ababa.

### What is known about this topic

- Hypertensive disorder during pregnancy can cause stillbirth;
- Being HIV infected during pregnancy is a predictor of stillbirth.

### What this study adds

- Pregnant women with non-cephalic foetal presentations during last ANC visits of the index pregnancy were three times more at risk of experiencing intrapartum stillbirth;
- Women with one or more alive children were proportionally less likely to experience intrapartum stillbirth compared to women without any child.

## References

[1] Fraser Diane M, Margaret A Cooper (1063). Myles' Textbook for Midwives.

[2] Frøen JF, Cacciatore J, McClure EM, Kuti O, Jokhio AH, Islam M, Shiffman J (2011). Stillbirths: why they matter. The Lancet.

[3] Murguia-Peniche T, Illescas-Zarate D, Chico-Barba G, Bhutta ZA (2016). An ecological study of stillbirths in Mexico from 2000 to 2013. Bull World Health Organ.

[4] Lawn JE, Blencowe H, Pattinson R, Cousens S, Kumar R, Ibiebele I, Gardosi J, Day LT, Stanton C (2011). Stillbirths: Where? When? Why? How to make the data count?. The Lancet.

[5] Goldenberg RL, Kirby R, Culhane JF (2004). Stillbirth: a review. J Matern Fetal Neonatal Med.

[6] Joy EL, Michael GG, Toni MN, Craig ER, Cynthia S (2010). Global report on preterm birth and stillbirth: definitions, description of the burden and opportunities to improve data. BMC Pregnancy and Childbirth.

[7] (2011). Central Statistics Agency. Ethiopian Demographic and Health Survey. Addis Ababa.

[8] Marshall J, Raynor M (2014). Myles Textbook for Midwives.

[9] Beauclair R, Petro G, Myer L (2014). The association between timing of initiation of antenatal care and stillbirths: a retrospective cohort study of pregnant women in Cape Town, South Africa. BMC Pregnancy Childbirth.

[10] Desire DT, Julia B (2016). Modifiable antenatal risk factors for stillbirth amongst pregnant women in the Omusati region, Namibia. African Journal of Primary Health Care & Family Medicine.

[11] Endris M, Deressa T, Belyhun Y, Moges F (2015). Seroprevalence of syphilis and human immunodeficiency virus infections among pregnant women who attend the University of Gondar teaching hospital, North-West Ethiopia: a cross sectional study. BMC Infect Dis.

[12] Federal Democratic Republic of Ethiopia Ministry of Health Health and Health related indicators.

[13] Cheptum JJ, Muiruri N, Mutua E, Gitonga M, Juma M (2016). Correlates of Stillbirths at Nyeri Provincial General Hospital, Kenya, 2009-2013: a retrospective study. International Journal of MCH and AIDS.

[14] Sharma S, Sidhu H, Kaur S (2016). Analytical study of intrauterine fetal death cases and associated maternal conditions. Int J Appl Basic Med Res.

[15] Ahmad AS, Samuelsen SO (2012). Hypertensive disorders in pregnancy and fetal death at different gestational lengths. A population study of 2, 121, 371 pregnancies. BJOG.

[16] Kilewo C, Natchu U, Young A, Donnell D, Brown E, Read J, Sharma U, Chi B, Goldenberg R, Hoffman I (2009). Hypertension in Pregnancy among HIV-Infected Women in Sub-Saharan Africa: Prevalence and Infant Outcomes. Afr J Reprod Health.

[17] Singh R, Prasad N, Banka A, Gupta A, Bhadauria D, Sharma RK, Kaul A (2015). Pregnancy in patients with chronic kidney disease: maternal and fetal outcomes. Indian J Nephrol.

[18] Trudell AS, Tuuli MG, Colditz GA, Macones GA, Odibo AO (2017). A stillbirth calculator: development and internal validation of a clinical prediction model to quantify stillbirth risk. PLoS One.

[19] Mwanri AW, Kinabo J, Ramaiya K, Feskens EJ (2015). Gestational diabetes mellitus in sub-Saharan Africa: systematic review and metaregression on prevalence and risk factors. Trop Med Int Health.

[20] Molla M (2015). Systematic Reviews of Prevalence and Associated Factors of Hypertension in Ethiopia: Finding the Evidence. Science Journal of Public Health.

[21] Sama CB, Feteh VF, Tindong M, Tanyi JT, Bihle NM, Angwafo FF (2017). Prevalence of maternal HIV infection and knowledge on mother-to-child transmission of HIV and its prevention among antenatal care attendees in a rural area in northwest Cameroon. PLoS One.

[22] Turnbull E, Lembalemba MK, Guffey MB, Bolton-Moore C, Mubiana-Mbewe M, Chintu N, Giganti MJ, Nalubamba-Phiri M, Stringer EM, Stringer JS (2011). Causes of stillbirth, neonatal death and early childhood death in rural Zambia by verbal autopsy assessments. Trop Med Int Health.

[23] Kim H-Y, Kasonde P, Mwiya M, Thea DM, Kankasa C, Moses S, Aldrovandi G, Kuhn L (2012). Pregnancy loss and role of infant HIV status on perinatal mortality among HIV-infected women. BMC Pediatrics.

[24] McClure EM, Goldenberg RL (2009). Infection and stillbirth. Semin Fetal Neonatal Med.

[25] Ferreira J, Borowski D, Czuba B, Cnota W, Wloch A, Sodowski K, Wielgos M, Wegrzyn P (2015). The evolution of fetal presentation during pregnancy: a retrospective, descriptive cross-sectional study. Acta Obstet Gynecol Scand.

[26] Kozuki N, Katz J, Khatry SK, Tielsch JM, LeClerq SC, Mullany LC (2017). Risk and burden of adverse intrapartum-related outcomes associated with non-cephalic and multiple birth in rural Nepal: a prospective cohort study. BMJ Open.

[27] Natasha N, Christine LR, Carolyn AC, Emily CO (2006). Diagnostic accuracy of clinical examination for detection of non-cephalic presentation in late pregnancy: cross sectional analytic study. BMJ.

[28] Afulani PA (2016). Determinants of stillbirths in Ghana: does quality of antenatal care matter?. BMC Pregnancy Childbirth.

[29] Hu IJ, Chen PC, Jeng SF, Hsieh CJ, Liao HF, Su YN, Lin SJ, Hsieh WS (2012). A nationwide survey of risk factors for stillbirth in Taiwan, 2001-2004. Pediatr Neonatol.

[30] Cheong-See F, Schuit E, Arroyo-Manzano D, Khalil A, Barrett J, Joseph KS, Asztalos E, Hack K, Lewi L, Lim A (2016). Prospective risk of stillbirth and neonatal complications in twin pregnancies: systematic review and meta-analysis. BMJ.

